# Clinical and molecular cytogenetic analyses of four families with 1q21.1 microdeletion or microduplication

**DOI:** 10.1002/jgm.2948

**Published:** 2017-04-21

**Authors:** Hong‐Dan Wang, Lin Liu, Dong Wu, Tao Li, Cun‐Ying Cui, Lian‐Zhong Zhang, Cheng‐Zeng Wang

**Affiliations:** ^1^ Institute of Medical Genetics, Henan Provincial People's HospitalZhengzhou University People's HospitalZhengzhouChina; ^2^ Department of Cardiovascular Ultrasound, Henan Provincial People's HospitalZhengzhou University People's HospitalZhengzhouChina; ^3^ Department of Ultrasound, the Affiliated Cancer HospitalZhengzhou UniversityZhengzhouChina

**Keywords:** 1q21.1 microdeletion, 1q21.1 microduplication, array‐comparative genomic hybridization, copy number variations

## Abstract

**Background:**

Little information is available regarding the penetrance of 1q21.1 copy number variants (CNVs). In the present study, we explored the clinical significance of 1q21.1 microdeletion or microduplication.

**Methods:**

In four families, chromosome karyotype was analyzed using G‐banding karyotype analysis technology. CNVs were detected using array‐comparative genomic hybridization (aCGH) and then a quantitative polymerase chain reaction (qPCR) was used to validate candidate CNVs. Sequence signature in the breakpoint region was analyzed using University of California Santa Cruz (UCSC) databases.

**Results:**

Except for karyotype 45, XX, der (13, 14) (q10, q10) in the mother (I2) of family 2, the karyotype was normal in all other members of the four families. In the mother (I2) and fetus (II2) of family 1, in newborn (II1) of family 2 and in fetus (II1) of family 3, there was 1.22‐Mb heterozygous microdeletion in the chromosome 1q21.1q21.2 region. The child (II1) of family 4 had a 1.46‐Mb heterozygous microduplication in the chromosome 1q21.1q21.2 region. The results of the qPCR were consistent with that of aCGH. There was large number of low copy repeats (LCRs) in the breakpoint region found by analysis of the UCSC database, and multiple LCRs were matched with sequences in the chromosome 1 short‐arm region.

**Conclusions:**

1q21.1 microdeletion and microduplication exhibit a variety of clinical manifestations and the specificity of their clinical features is not high. The penetrance of the distal 1q21.1 microdeletion may be affected by other factors in the present study. In summary, we report the discovery of a new distal 1q21.1 microduplication, which enriches the CNV spectrum in the 1q21.1 region and is conducive to prenatal genetic counseling.

## 1 INTRODUCTION

The chromosome 1q21.1 locus is a complex region with multiple low‐copy repeats (LCRs) that make the region susceptible to recurrent deletions and duplications. This may result in the susceptibility of this region to both pathological and nonpathological copy number variants (CNVs). The chromosome 1q21.1 region can be subdivided into two distinctive regions. The proximal region, extending from breakpoint (BPs) 2 to BP3, spans approximately 200 kb (chr1: 145.4–145.6 Mb, GRCh37/hg19) and the distal region, extending from BP3 to BP4, spans 1.35 Mb (chr1: 146.5–147.9 Mb, GRCh37/h19).^1^, ^2^

Individuals with 1q21.1 recurrent microdeletion may have a wide range of clinical manifestations. The most common findings include mildly dysmorphic facies and developmental delay. However, there is no clinically recognizable syndrome, and some individuals with this microdeletion do not present obvious clinical findings.^3^, ^4^, ^5^ The 1q21.1 recurrent microdeletion is inherited in an autosomal teddominant manner, with 18–50% of deletions occurring *de novo* and 50–82% being inherited from their parents. No genotype–phenotype correlations are observed in those with the 1q21.1 recurrent microdeletion. Little information is available regarding penetrance of the 1q21.1 recurrent microdeletion. Similar to several other recurrent microdeletions (e.g. 16p11.2, 15q13.3), the 1q21.1 recurrent microdeletion can be inherited from the parents with minimally abnormal or completely normal clinical findings. In addition, several relatives of probands with the same 1q21.1 microdeletion have a normal phenotype or only mild manifestations.^6^, ^7^ Because the number of individuals published to date is limited, the exact phenotypic consequences remain unclear. In the present study, we describe the clinical phenotype and molecular cytogenetics of three families with 1q21.1 microdeletions.

1q21.1 duplication syndrome is a rare aberration of chromosome 1 with multiple congenital malformations, including developmental delay, autism spectrum disorder, dysmorphic features and congenital heart anomalies. Congenital heart malformations occur in approximately 18% and 29% of patients with proximal and distal 1q21.1 microduplications, respectively. These comprise a broad spectrum of abnormalities, including left‐sided, right‐sided, conotruncal and septal defects.^8^ In the present study, we describe the clinical phenotype and molecular cytogenetics of one family with 1q21.1 microduplication. The distal 1q21.1 microduplication was discovered for the first time in the present study, enriching the CNV spectrum in the 1q21.1 region and providing a basis for clinic and prenatal genetic counseling.

## 2 MATERIALS AND METHODS

All study methods were approved by the Ethics Committee of Henan Provincial Peoples Hospital. Written informed consent was provided by all subjects who enrolled in the study, as well as their parents.

### 2.1 Subjects

**Figure 1:**
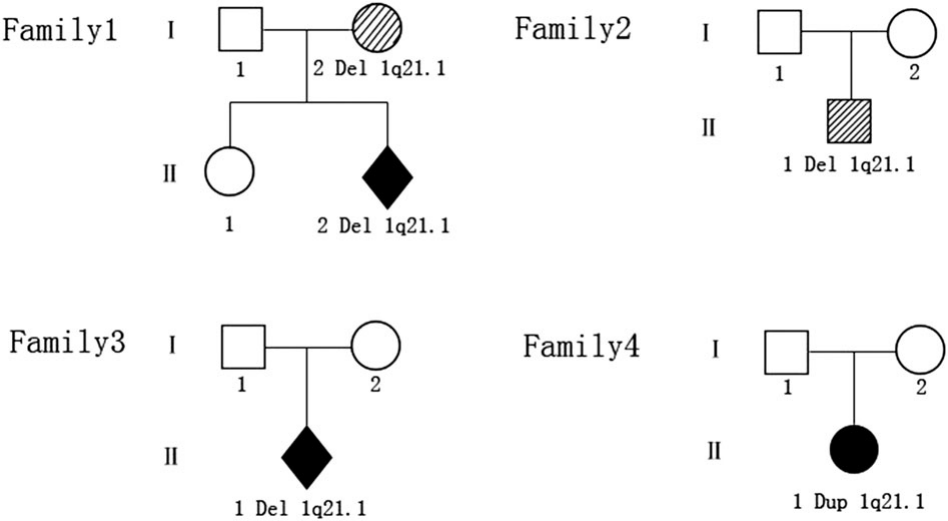
Genealogical tree for the four families in this study. I, parent; II, offspring; □, male; ○, female; ⋄, fetus; black, CNV patient with clinical symptoms; oblique line, CNV patient without clinical symptoms

Four participants including two fetuses with malformations, a child with Tetralogy of Fallot and a normal newborn, were from four Chinese families without a family history of congenital malformation. Parents of these patients went to the Medical Genetics Institute for genetic counseling. A routine clinical examination was performed. Detailed birth, medical data and clinical manifestations were collected. The pedigree of four families is shown in Figure 1.

### 2.2 G‐banding karyotype analysis

Amniotic fluid (10 ml) and venous blood (3 ml) were collected from two fetuses and other members of the four families, respectively. Amniotic fluid (1 ml) or venous blood (1 ml) was inoculated into RPMI1640 at 37°C for 72 h. Colchicine was added 1 h before collecting samples. The chromosomes were prepared using the routine method and then underwent Giemsa staining followed by analysis of 30 mitotic phases under a microscope using a fully automatic karyotype analysis system (Leica Microsystems, Wetzlar, Germany). The karyotype was described based on the International Naming System of Human Cell Genetics (ISCN2013).

### 2.3 DNA extraction

DNA was extracted using column whole blood/tissue genomic DNA extraction kit (TianGen Biochemical Science and Technology Co., Ltd, Beijing, China) in accordance with the manufacturer's instructions. DNA concentration and purity were determined using an ultra‐microspectrophotometer (NanoDrop 2000; NanoDrop, Wilmington, DE, USA) and the results obtained indicated that the *A*_260_/*A*_280_ ratios of all DNA samples were between 1.80 and 1.90. The DNA concentration was determined using Qubit quantitative platform (Qubit 2.0; Thermo Fisher Scientific Inc., Waltham, MA, USA).

### 2.4 Array‐comparative genomic hybridization (aCGH)

DNA quality was checked and the qualified DNA samples were then detected using SurePrint G3 Human CGH Microarray 8 × 60 K chips (Agilent Technologies Inc., Santa Clara, CA, USA). After lysis, labeling Cy‐dUTP and Cy‐dUTP, purification, hybridization and washing, scanning and data extraction were performed using a Microarray scanner (Agilent Technologies Inc.) and relevant software that could indicate CNVs with three consecutive probe log_2_ values greater than 0.25 or less than −0.25. Most of pathogenic CNVs (99.34%) are larger than 300 kb.^9^ CNVs greater than 200 kb are generally detected.^10^ In the present study, CNVs with chromosomal aneuploidy and greater than 200 kb were detected, and so the possibility of minor anomalies occurring in chromosome structures or gene fragment was not excluded. Chip sequence information was from hg19. The microarray results were further compared with the University of California Santa Cruz (UCSC), Database of Chromosomal Imbalance and Phenotype in Humans using Ensembl Resources (DECIPHER), Database of Genomic Variants (DGV), Institute of Singapore Chartered Accountants (ISCA) and Online Mendelian Inheritance in Man (OMIM) databases to analyze the pathogenicity of CNVs.

### 2.5 Quantitative polymerase chain reaction (qPCR)

Microdeletions and microduplications detected by aCGH were validated using a StepOne type fluorescent quantitative PCR instrument (Applied Biosystems, Foster City, CA, USA). The primers used in the qPCR are shown in the Supporting infromation (Table S1). GADPH served as a reference gene.

### 2.6 Sequence analysis of the breakpoint region

The sequence signature in the breakpoint region was analyzed using UCSC databases, as well as National Center for Biotechnology Information (NCBI).

## 3 RESULTS

**Figure 2:**
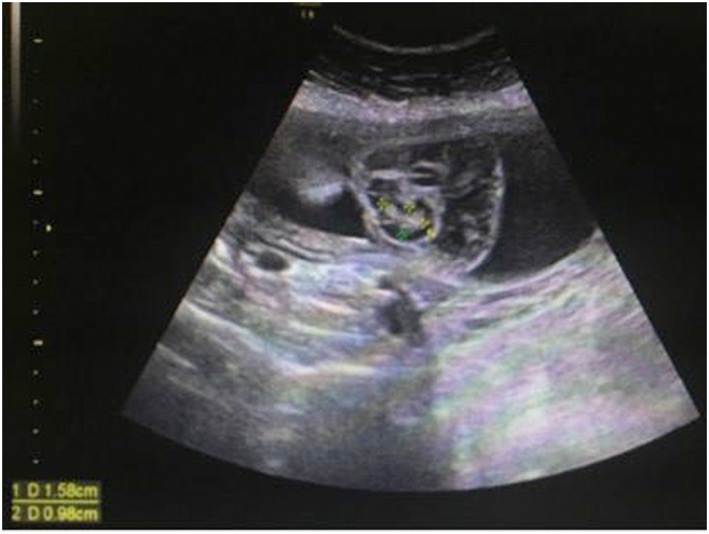
Fetal encephalomeningocele. Behind the fetal head, there is a 56 × 52 mm fluid sonolucent area that is multilocular as a result of the existence of septations with a 15 × 9 mm high‐level echo

**Figure 3:**
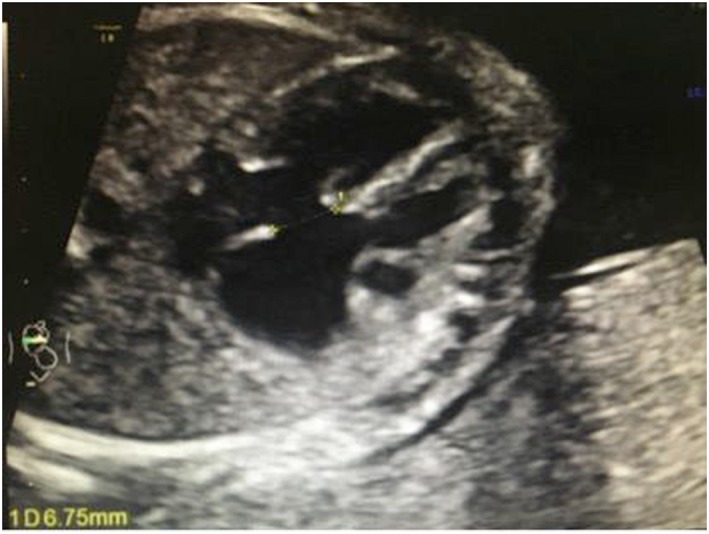
The fetus has complete atrioventricular septal defect. The four‐chamber view of the fetus shows the disappearance of cross section with a 6.75‐mm defect

**Figure 4:**
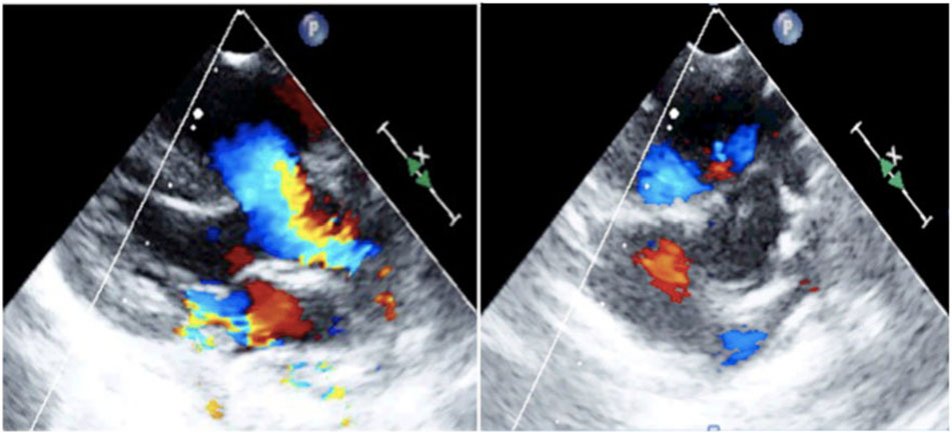
Tetralogy of Fallot combined with acleistocardia. Left ventricular long‐axis view shows the ventricular septal defect and aortic over‐riding (left). Large arterial short‐axis view shows the main pulmonary artery, left pulmonary artery, right pulmonary artery stenosis and the increased thickness of the right ventricular anterior wall (right)

In family 1, prenatal diagnosis and assay of genomic CNVs were required because the type‐B ultrasonic instrument showed encephalomeningocele (Figure 2).in the fetus (II2). In family 2, karyotype analysis and assay of genomic CNVs were required because the mother (I2) had karyotypic abnormality. In family 3, prenatal diagnosis and assay of genomic CNVs were required because the type‐B ultrasonic instrument showed complete atrioventricular septal defect (Figure 3).in the fetus (II1). In family 4, karyotype analysis and assay of genomic CNVs were required because there were the symptoms of dyspnea and cyanosis, systolic ejection murmurs were available beside the left sternal border and between the second and fourth ribs, and the type‐B ultrasonic instrument showed tetralogy of Fallot combined with acleistocardia (Figure 4) in the child (II1). All members of the four families were followed up and their clinical data were collected (Table 1).

**Table 1:** Clinical data of all members in the four families

	Case	Sex	Age	CNVs	Genetic mode	Karyotypic abnormalities	Brain abnormalities	Cardiovascular abnormalities
Family 1	I1	M	25Y	No	–	No	No	No
	I2	F	26Y	Del 1q21.1	Unknown	No	No	No
	II1	F	6Y	No	–	No	No	No
	II2	F	TOP	Del 1q21.1	Maternally inherited	No	Encephalomeningocele	No
Family 2	I1	M	31Y	No	–	No	No	No
	I2	F	31Y	No	–	45, XX, der (13, 14)( q10,q10)	No	No
	II1	M	25D	Del 1q21.1	*de novo*	No	No	No
Family 3	I1	M	27Y	No	–	No	No	No
	I2	F	28Y	No	–	No	No	No
	II1	M	TOP	Del 1q21.1	*de novo*	No	No	Complete atrioventricular septal defect
Family 4	I1	M	32Y	No	–	No	No	No
	I2	F	30Y	No	–	No	No	No
	II1	F	9M6D	Dup 1q21.1	*de novo*	No	No	Tetralogy of Fallot combined with acleistocardia

CNVs, copy number variants; F, female; M, male; TOP, termination of pregnancy.

**Figure 5:**
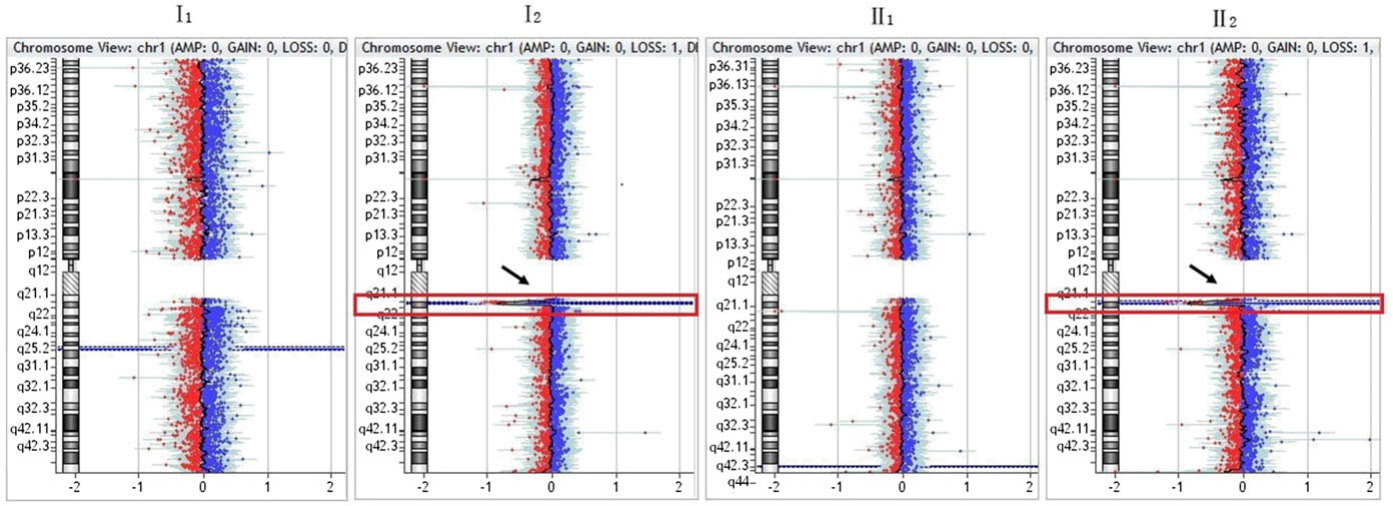
Results of array comparative genomic hybridization in family 1. As indicated by the arrows, there was the same 1.22‐Mb heterozygous microdeletion with the molecular karyotype of arr[hg19] 1q21.1q21.2(146,564,743–147,786,706) × 1, respectively, in the mother (I2) and fetus (II2)

**Figure 6:**
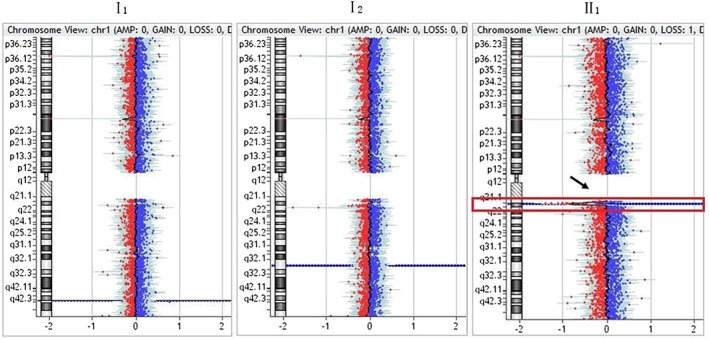
Results of array comparative genomic hybridization in family 2. As indicated by the arrow, there was a *de novo* 1.22‐Mb heterozygous microdeletion with the molecular karyotype of arr[hg19] 1q21.1q21.2(146,564,743–147,786,706) × 1 in the newborn (II1)

**Figure 7:**
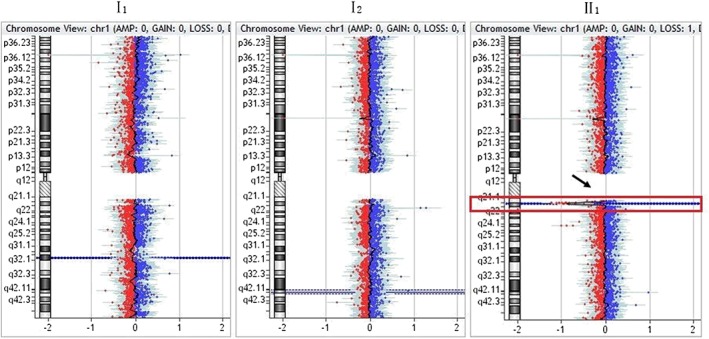
Results of array comparative genomic hybridization in family 3. As indicated by the arrow, there was a *de novo* 1.22‐Mb heterozygous microdeletion with the molecular karyotype of arr[hg19] 1q21.1q21.2(146,564,743–147,786,706) × 1 in the fetus (II1)

Except for karyotype 45, XX, der (13, 14) (q10, q10) in the mother (I2) of family 2, the karyotype was normal in all other members of the four families. aCGH indicated a 1.22‐Mb heterozygous microdeletion with the karyotype of arr[hg19] 1q21.1q21.2(146,564,743–147,786,706) × 1, respectively in the mother (I2) and fetus (II2) of family 1 (Figure 5). in newborn (II1) of family 2 (Figure 6). and in fetus (II1) of family 3 (Figure 7). qPCR showed that the copy number of this fragment‐related gene was 0.5 times the copy number of the control, suggesting that the qPCR result was consistent with that of aCGH. The 1q21.1q21.2 region of chromosome 1 with a 1.22‐Mb microdeletion contains 14 genes, including *FMO5*, *CHD1L*, *BCL9*, *GJA5*, *GJA8*, *GPR89B*, *LOC728989*, *PRKAB2*, *PDIA3P*, *ACP6*, *GPR89C*, *PDZK1P1*, *NBPF11* and *NBPF24*.

**Figure 8:**
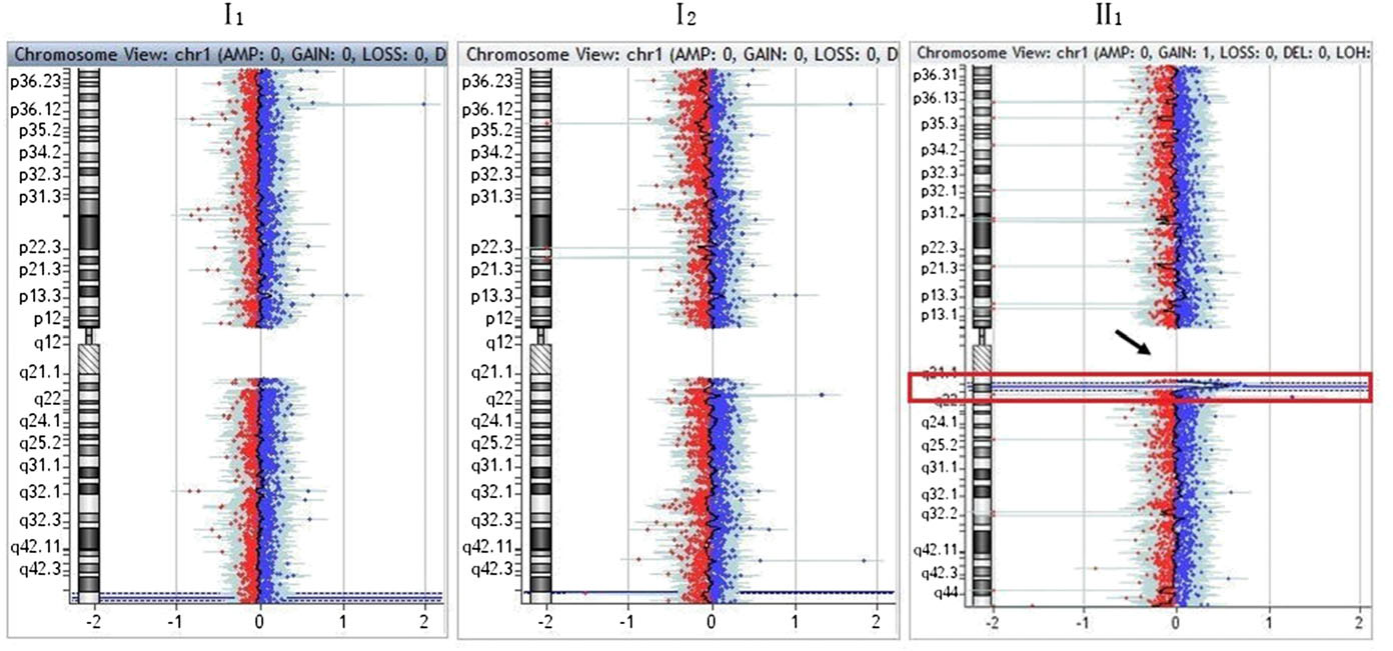
Results of array comparative genomic hybridization in family 4. As indicated by the arrow, there was a *de novo* 1.46‐Mb heterozygous microduplication with the molecular karyotype of arr[hg19] 1q21.1q21.2: (146,324,068–147,786,706) × 3 in the child (II1)

aCGH indicated a 1.46‐Mb heterozygous microduplication with the karyotype of arr[hg19] 1q21.1q21.2: (146,324,068–147,786,706) × 3 in the child (II1) of family 4 (Figure 8). qPCR showed that the copy number of this fragment‐related gene was 1.5 times the copy number of the control, suggesting that the qPCR result was consistent with that of aCGH. The 1q21.1q21.2 region of chromosome 1 with a 1.46‐Mb microduplication contains 14 genes, including *FMO5*, *CHD1L*, *BCL9*, *GJA5*, *GJA8*, *GPR89B*, *LOC728989*, *PRKAB2*, *PDIA3P*, *ACP6*, *GPR89C*, *PDZK1P1*, *NBPF11* and *NBPF24*.

**Figure 9:**
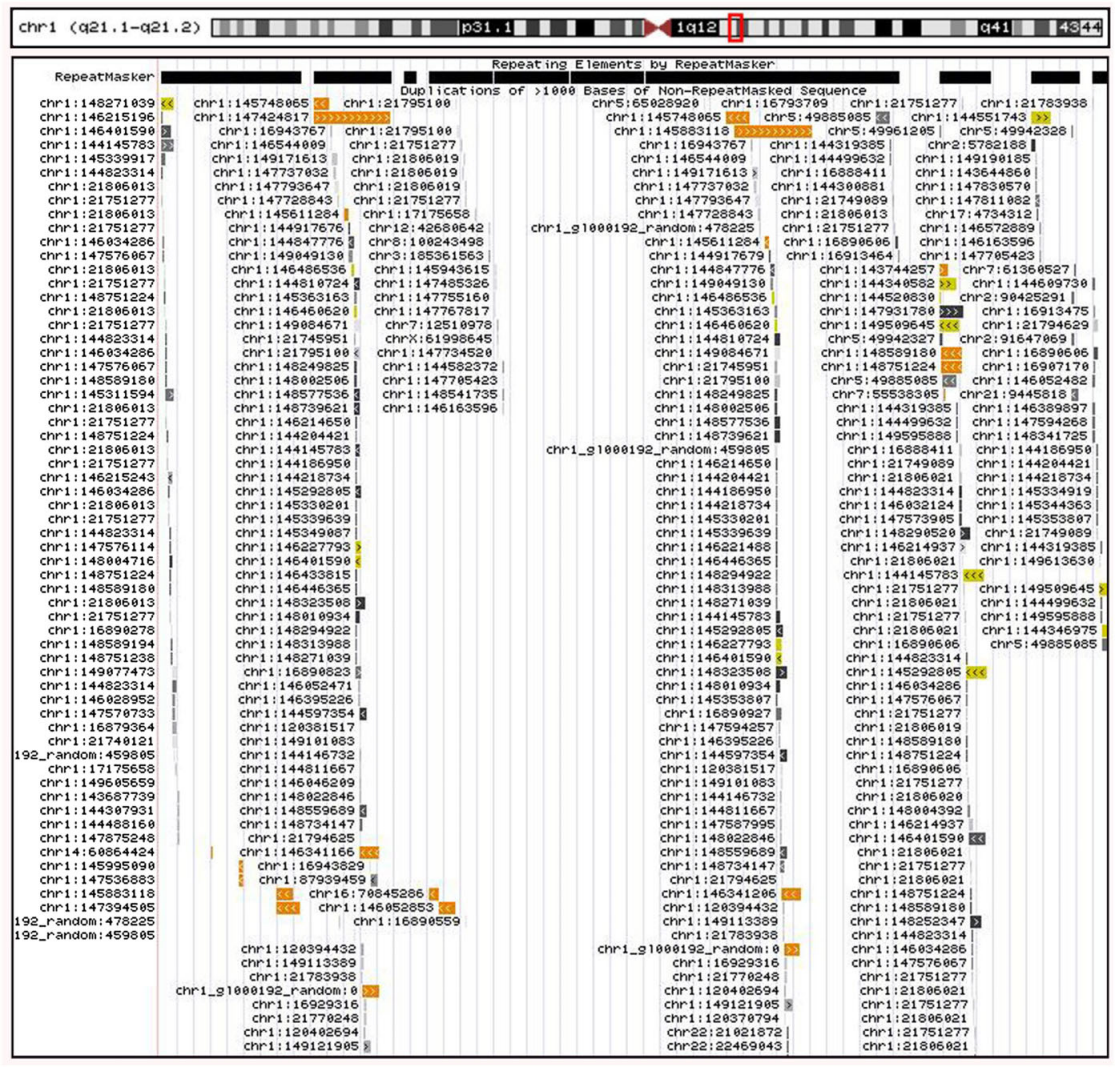
The UCSC database indicates a large number of repeated similar fragments in the 1q21.1q21.2 region with CNVs

The UCSC database indicates that there a large number of similar repeated fragments in this breakpoint region (Figure 9). There was a large number of LCRs or segmental duplications in the breakpoint region and multiple LCRs were matched with the sequences in the chromosome 1 short‐arm region. For example, the sequence matching reached 99.94% between the chr1:145883119–146164650 region and the chr1:147424818–147706477 region, 99.12% between the chr1:145748066–145833117 region and the chr1:147394506–147482095 region, 98.19% between the chr1:145292806–145368562 region and the chr1:148271040–148347356 region, 90.51% between the chr1:146544010–146552754 region and the chr1:147485327–147487338 region, and so on. Utilizing these LCRs copies in cis as recombination substrates for ectopic cross‐overs, these LCRs may mediate nonallelic homologous recombination (NAHR), leading to recurrent genomic deletions and reciprocal duplications. In other words, NAHR events in trans between LCRs on nonhomologous chromosomes can cause recurrent constitutional translocations and lead to CNVs.

## 4 DISCUSSION

Copy number polymorphisms (CNPs) are also called CNVs. CNVs widely exist in the human genome. CNVs refer to 1‐kb to several Mb genome rearrangements, such as deletion, duplication, insertion and complex multipoint variations, which account for approximately 12% of human genome DNA.^11^ Generally, CNVs do not contain the insertion and complex multipoint variations. CNVs mainly refer to genome duplication or deletion. CNVs can occur not only in an individual gene, but also in a continuous suite of genes. Therefore, the number of mononucleotide affected by CNVs is much more than that affected by a single nucleotide polymorphism, such that CNVs are one of the important pathogenic factors for humans.^11^, ^12^, ^13^, ^14^ In recent years, it has been reported that the CNV range is related to human phenotype diversity and susceptibility to certain diseases, the incidence of disease as a result of CNVs is much higher than that caused a by single nucleotide polymorphism, and pathogenic CNVs are very different from nonpathogenic CNVs.^15^, ^16^

aCGH technology, comprising molecular karyotype analysis, can detect chromosomal unbalanced CNVs genomewide, especially microdeletions and microduplications. In 2010, the International Standards for Cytogenomic Arrays Consortium (ISCA Consortium) recommended aCGH as a clinical first‐line detection method for agnogenic developmental delay, mental retardation, malformations with different signs and autism.^10^

1q21.1 CNV (OMIM 612474 and 612475) is associated with a highly variable phenotype.^5^, ^17^, ^18^, ^19^, ^20^, ^21^, ^22^ It is difficult to evaluate the clinical significance of this CNV. CNVs occurring in the 1q21.1 region include microdeletion and microduplication, which exhibit a variety of clinical manifestations. 1q21.1 microdeletion may manifest delayed growth, microcephaly, abnormal facies, cataracts and congenital heart disease, whereas 1q21.1 microduplication usually exhibits mental retardation, autism and megalencephalon. However, interindividual clinical variability, incomplete penetrance and a lack of specific facial features may result in different manifestations between individuals. Congenital heart disease with different anatomical features, including left ventricular outflow tract obstruction, conus arteriosus and septal defects, has been found in patients with 1q21.1 deletion syndrome. In addition, a nonsyndromic congenital heart defect was found in both patients with 1q21.1 microdeletion and in patients with 1q21.1 microduplication.^5^, ^17^, ^18^, ^19^, ^20^, ^21^, ^22^ In the present study, patients with 1q21.1 microdeletion manifested normal, meningoencephalocele or complete atrial septal defects. Meningoencephalocele has not been reported, although complete atrial septal defects have been reported in patients with the 1q21.1 microdeletion. The normal phenotype in the patient with a 1q21.1 microdeletion in the present study suggests that the phenotype of the patients with 1q21.1 is associated with interindividual clinical variability, incomplete penetrance and a lack of specific facial features. In the present study, a patient with 1q21.1 duplication had Tetralogy of Fallot and acleistocardia, which has not been described.

The chromosome 1q21.1 locus is a complex region with multiple LCRs that make the region susceptible to NAHR. NAHR can lead to recurrent genomic deletions and reciprocal duplications. Chromosomal band 1q21.1 can be divided into two distinct regions, proximal (BP2–BP3) and distal (BP3–BP4), based on LCRs that mediate recurrent rearrangements. Proximal microdeletions are known as a predisposing factor for thrombocytopenia‐absent radius syndrome.^23^, ^24^ The proximal 1q21.1 microdeletions and microduplications are associated with a failure to thrive, feeding problems, developmental delay and/or intellectual disability, behavior problems (including autism and attention deficit hyperactivity disorder), congenital heart malformations, and a variety of dysmorphic features.^25^

Microdeletions and microduplications of the proximal region within 1q21.1 have been more extensively studied than distal microdeletions and microduplications. Otherwise, the phenotypic features varied among individuals with these distal microdeletions and microduplications. The current literature demonstrates that the microdeletions and microduplications of the distal 1q21.1 region are associated with a variety of morbidities.^25^ Among the 13 members from four families in the present study, four members had the distal (BP3–BP4) 1q21.1 deletion and one had the distal (BP3–BP4) 1q21.1 duplication. Interestingly, the mother (I2) of family 1 and the newborn (II1) of family 2 had none of the clinical symptoms and other diseases reported above, although they all carried a distal 1q21.1 microdeletion. As proposed by Girirajan et al.,^26^ several other possibilities, such as epigenetic phenomena, expression or regulatory variation among genes in the vicinity of the unbalanced region, the unmasking of recessive alleles and the possibility of a ‘two‐hit’ model, may account for the phenotypic variability of some genomic diseases. The number of individuals published to date is limited, and the exact phenotypic consequences remain unclear.

1q21.1 duplication syndrome is a rare aberration of chromosome 1 and it has not been reported in China. In the present study, a distal 1.46‐Mb 1q21.1 microduplication was found in the child (II1) of family 4. The child, after birth, had dyspnea and cyanosis with B‐mode ultrasound of tetralogy of Fallot, although other members in family 4 were normal. Soemedi et al.^27^ observed 2436 cases with congenital heart disease and found that the 1q21.1 microduplication was common in the cases with tetralogy of Fallot. This is consistent with the results of the present study. It has been recently reported that tetralogy of Fallot is associated with the 1q21.1 region or *GJA5* gene mutation in the 1q21.1 region.^28^ The *GJA5* gene codes for a type of cardiac connexin‐40 that plays a key role in cell adhesion and communication between cells.^27^ Among tetralogy of Fallot‐related genes in the vicinity of chromosome 1, it has been confirmed that there is *GHD1L* over‐expression in the cases with tetralogy of Fallot, double‐outlet right ventricle or pulmonary artery stenosis.^27^ Helicase protein encoded by the *GHD1L* gene is involved in repair by transformation of ATP into poly ADP‐ribose, and is closely related to DNA repair after chromatin unwinding.^29^ Adenylate activating protease β2 subunit encoded by the *PRKAB2* gene is found to have high expression in the right ventricular outflow tract.^30^ Because there are very few related studies, the effects of other genes in this region on the development and differentiation of tissues and organs remain to be further explored.

In the present study, we investigated clinical data and genetics in the four families aiming to explore the relationship between 1q21.1 locus variation and diseases. 1q21.1 microdeletion and microduplication exhibit a variety of clinical manifestations and the specificity of their clinical features is not high. In prenatal diagnosis, if pathogenicity‐reported *de novo* CNVs are detected, it is necessary to evaluate fetal development using other prenatal diagnosis technology; if the CNVs inherited from parents are found, their pathogenicity is not completely denied because the pathogenic penetrance of these CNVs may be affected by other factors, and so it is also necessary to evaluate fetal development using other prenatal diagnosis technology. In addition, the present study discovered a new distal 1q21.1 microduplication that enriches the CNV spectrum in the 1q21.1 region. In summary, the present study provides molecular genetic data and is conducive to genetic counseling with respect to CNV‐related diseases.

## Supporting information


**Supplementary Table 1** The primers used in quantitative polymerase chain reaction.
