# Supplementary material for: Clinical and molecular cytogenetic analyses of four families with 1q21.1 microdeletion or microduplication
Source: J Gene Med. 2017 Apr 21;19(4):e2948. doi: 10.1002/jgm.2948 (PMC5413856; doi:10.1002/jgm.2948)
Supplement: Supplementary file 1 — Supplementary Table 1 The primers used in quantitative polymerase chain reaction. [file JGM-19-na-s001.doc]

**Supplementary Table 1** The primers used in quantitative polymerase chain reaction

| Primer name | Primers (5’-3’) | Annealing temperature (℃) | Amplicon size (bp) |
| --- | --- | --- | --- |
| FMO5-F | CTTTCTTTAGGACAGGCGACAC | 59.1 | 120 |
| FMO5-R | CTTCTACGCAGCACTTGATGG |
| GJA5-F | GCTCAAAGGCAACTCAGAACC | 58.6 | 104 |
| GJA5-R | GGAATACTGCGAGGGAGAATAA |
| PDZK1P1-F | TGAGTCTCTAGAAAAGTCAAGGATC | 58.5 | 170 |
| PDZK1P1-R | GCTAACAAAGCAACAGGCACT |
| GADPH-F | CTGGTCTGAGGTTAAATATAGCTGC | 57.5 | 112 |
| GADPH-R | AGGAGTGGGAGCACAGGTAAG |
